# A Biomechanical Comparison between Taylor’s Spatial Frame and Ilizarov External Fixator

**DOI:** 10.5704/MOJ.1407.012

**Published:** 2014-07

**Authors:** BB Tan, R Shanmugam, R Gunalan, YP Chua, G Hossain, A Saw

**Affiliations:** National Orthopaedic Centre of Excellence for Research and Learning (NOCERAL), University of Malaya, Kuala Lumpur; National Orthopaedic Centre of Excellence for Research and Learning (NOCERAL), University of Malaya, Kuala Lumpur; National Orthopaedic Centre of Excellence for Research and Learning (NOCERAL), University of Malaya, Kuala Lumpur; National Orthopaedic Centre of Excellence for Research and Learning (NOCERAL), University of Malaya, Kuala Lumpur; National Orthopaedic Centre of Excellence for Research and Learning (NOCERAL), University of Malaya, Kuala Lumpur; National Orthopaedic Centre of Excellence for Research and Learning (NOCERAL), University of Malaya, Kuala Lumpur

## Abstract

Taylor’s spatial frame (TSF) and Ilizarov external fixators (IEF) are two circular external fixator commonly used to address complex deformity and fractures. There is currently no data available comparing the biomechanical properties of these two external fixators. This study looks into the mechanical characteristics of each system. TSF rings with 6 oblique struts, 4 tube connectors, 4 threaded rods, and 6 threaded rods were compared to a standard IEF rings with 4 threaded rods. Compression and torsional loading was performed to the frame as well as construct with Polyvinylchloride tubes. TSF rings with 4 tube connectors had the highest stiffness (3288 N/mm) while TSF rings with 6 struts was the least stiff. The situation was reversed for torsion where TSF rings with 6 oblique struts had the highest torsional stiffness (82.01 Nm/Degree) and frame Ilizarov rings with 4 threaded rods the least. Standard TSF construct of two ring with 6 oblique struts have better torsional stiffness and lower axial stiffness compared to the standard IEF.

**Key Words:**

Taylor’s Spatial Frame, Ilizarov External Fixator, Biomechanical properties

## Introduction

Conventional Ilizarov external fixator (IEF) is composed of stainless steel rings connected with threaded rods that can be configured in various ways to Manage different indications. The frame is fixed to the bone using either stainless steel wires under tension or rigid Schanz pins that causes minimally disruption of the soft tissue. These basic Ilizarov external fixator construct and method of fixation provides favorable mechanical and biological environment for bone healing ^1, 2^. Accurate and purposeful positioning of hinges and distractors will also allow gradual correction of complex deformities.

Planning and application of Ilizarov external fixtor require considerable experience, and the need for post-operative frame re-adjustment is not infrequent. Newer generation of external fixators make use of hexapod system to perform gradual multiaxial correction in six degree of freedom without changing the position and orientation or connecting elements. In this type of fixator, six obliquely placed adjustable struts are connected to the proximal and distal rings. To achieve desirable correction of the bone segments, gradual adjustment of individual strut lengths will be guided by computer software.

Stiffness and fixation stability of conventional IEF have been reported in literature ^3-5^. We would expect multiaxial external fixator frame to have additional free play that arises from the universal hinges on both sides of the oblique struts. To our knowledge, there has been no study comparing the mechanical properties of the conventional IEF with multiaxial frame. Information on the mechanical properties may allow the user to modify the construct of these frames to provide optimum environment for fracture union or bone healing.

We therefore conducted this study to compare the mechanical properties of IEF and multiaxial external fixator.

## Materials and Method

**Fig. 1: Schematic representation of the frame designs used in the study.**
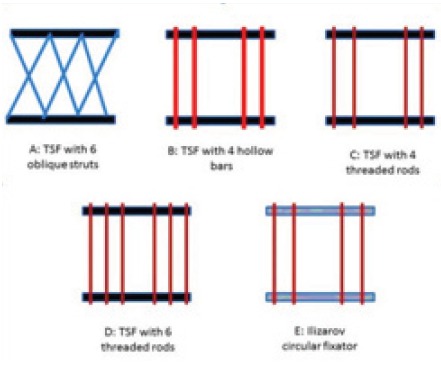


Four pairs of 155mm Taylor Spatial Frame (TSF, Smith and Nephew, Memphis, USA) full rings and one pair of 150mm IEF rings (Smith and Nephew, Memphis, USA) were used in this study. Five frame configurations were constructed (Fig. 1). Frame A : TSF rings with 6 oblique struts (medium Fastfix fixation struts), Frame B : TSF rings with four hollow stainless steel bars (outer diameter 10mm and inner diameter 6mm), Frame C : TSF rings with four threaded rods (8mm adult Ilizarov threaded rods), Frame D : TSF rings with six threaded rods (8mm adult Ilizarov threaded rods) and Frame E : IEF rings with 4 threaded rods (8mm adult Ilizarov threaded rods). Distance between the rings was fixed 200mm for all the five constructs.

**Fig. 2a 2b: 2a - Loading test on Frame A along the midaxial line. 2b - Offset axial loading on Frame C.**
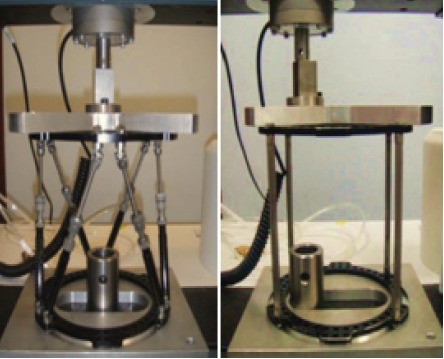


These frames were loaded on an Instron 3365 (MA, USA) under displacement control with constant ram speed of 0.5mm/sec up to a maximum load of 700 N. The load of 700N was chosen as this is the average weight of adult patients ^6,7^. Loading was performed along the midaxial plane as well as offset axial loading (Fig. 2). Load displacement curves were plotted from the data to calculate the stiffness of the frames. The loadings were repeated six times to obtain an average value for comparison. Data from a pilot study conducted earlier showed that the loading was within the elastic region of the frame, thus the same frame was reused for subsequent tests.

Torsion load was applied manually using a torque wrench and measuring the angular displacement between the proximal and distal rings. An incremental load of 5Nm up to a maximum of 30 Nm of torque was applied ^8^. The above measurement was repeated six times to obtain an average value. Torque vs angular displacement curves were plotted to calculate the torsional stiffness.

The same construct were later mounted with polyvinylchloride (PVC) tubes (inner diameter of 42mm and outer diameter of 32mm) to represent bone inner diameter of 42 mm and outer diameter of 32 mm PVC was used to standardize the material property as the aim of this study is to compare the stiffness of the frame construct and not the holding strength to the bone. The other consideration is that the PVC tubes are readily available and cheap compared to cadaveric bone. There has been studies in the past utilizing PVC and wood to simulate bone 9,10. The tubes were fixed to the frames eccentric to center of the ring to simulate tibia bone fixation in clinical practice. One surface of the tube will be placed 20mm from the inner border of the ring to represent the anterior medial cortex of tibia bone (Fig. 3). For wire fixation, 1.5mm drill bit was used to create a initial hole before 1.8mm stainless steel Ilizarov wire was inserted and tensioned to 1100N using a wire tensioning device (Smith and Nephew, Memphis, USA). The angle between the wires was standardized to 60o based of literature review 4,8,11. Two 5.0mm Schanz pins were then inserted from the anteromedial aspect above and below the rings using a three-hole rancho cube for each pin. The final construct had a 20mm gap between the PVC tubes to ensure that the entire load was transferred through the fixator frame (Fig. 4). Frames with the PVC tubes were loaded in axial and four bending modes (anterior, posterior, medial and lateral) using the same setting as for the frame only. Four bending loads were applied to the PVC tubes with eccentric placement within the ring to test the bending stiffness of the constructs in clinical practice. Finally torsional load was applied. Loading was applied using a custom made jig and the displacement at the fracture site was measured using a digital caliper with an accuracy of two decimal points.

The first author performed all the mechanical testing and its measurement. Analyses of the results were performed with SPSS ver 17.0 and Microsoft Excel 2010, using analysis of variance (ANOVA). P value of 7lt; 0.05 was considered significant.

## RESULTS

**Fig. 5: Graph showing the respective axial, offset axial and torsion stiffness of each frame design.**
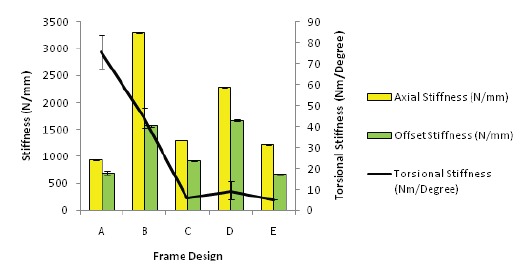


When we compare the frame stiffness, true axial loading showed higher stiffness values compared to offset axial loading (Fig. 5). Frame B (TSF rings with hollow steel bars) recorded the highest stiffness value of 3288 N/m that is statistically significant (p < 0.05) compared to all other constructs. Frames B,C,D.E were significantly stiffer than Frame A (TSF rings with 6 oblique struts, p<0.05). However, in torsional loading, Frame A recorded the highest torsional stiffness of 82.01 Nm/ degree, and this was significantly stiffer compared to Frame E and Frame C (TSF and IEF rings with 4 threaded rods). Differences with Frames B and D (TSF rings with hollow rods and 6 threaded rods) were not statistically significant.

When we compare the loading stiffness through the bone substitute, the overall pattern is similar to the loading of the frame with Frame B (TSF rings with hollow rods) being the stiffest in axial loading while the Frame A (TSF rings with 6 struts) was stiffest in torsional loading (Fig. 6). However, there was no significance difference between the Frame A (TSF with 6 struts) with any other Frames (C,D,E), except with Frame B in medial and lateral offset bending (with frame B being stiffer with a value of 135.5 Nm/mm). Torsional loading showed that Frame A (TSF rings with 6 struts) to be significantly stiffer compared to Frame E (Ilizarov rings with 4 threaded rods, p < 0.001).

**Fig. 7: Graph showing comparison between the stiffness for the frame and tube in both axial and torsional loading.**
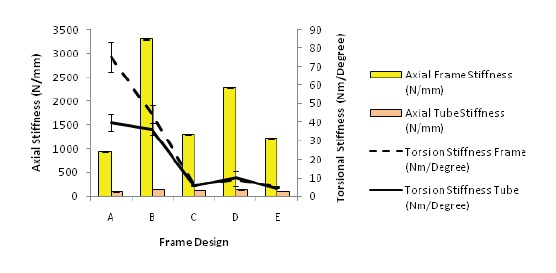


Axial stiffness reduces markedly when we compared testing of the frames and testing of the tubes. However, for torsional stiffness, the differences between frame and tube testing were less obvious (Fig. 7).

## Discussion

Bone is a living and dynamic entity and bone healing process is influenced by both biology as well as mechanical environment. Preservation of blood supply to the bone provides a favorable biological condition for healing to occur. At the same time, adequate stability of bone ends is also essential to prevent excessive movement that may lead to nonunion. It has been reported that interfragmentary strain of less than 10% between bone ends was necessary for desirable fracture union ^12^. Ilizarov in his experiments with canines reported a direct correlation between frame stiffness and bone regeneration^13^. External fixator naturally would not be as stiff as internal fixation due to the long lever arm of the fixation wires and pins. This was also evident from our study where stiffness of the frame was much higher compared to that of the bone substitutes especially on axial loading (Fig. 7).

When we analyze the stiffness on axial loading, our results showed that TSF rings are stiffer than Ilizarov rings as evidenced by the higher stiffness of Frame C compared to Frame E, although the difference was not statistically significant. TSF rings (made from Aluminium alloy) are thicker compared to IEF rings (made from medical grade stainless steel), and this may be the main contributing factor. However, when we compared the standard TSF configuration with 6 oblique struts to the standard IEF configuration with 4 threaded rods, we noted that IEF (Frame E) was significantly stiffer than TSF frame (Frame A) on axial loading. Lower stiffness recorded on TSF frame was mainly contributed by the design and configuration of the connecting struts. As expected, stiffness of PVC tubes fixed with both types of fixator frames will be lower than stiffness of corresponding fixator frames alone, due to the long lever arm of wires and pins used to secure the bone substitutes on to the frames. Degree of stiffness on the PVC tubes fixed with TSF frame remained lower than that of IEF frame, but the difference was not statistically significant.

When we compared torsional stiffness of the frames, TSF frame (Frame A) stands out as the frame with highest stiffness compared to other types of frames (Frames B,C,D,E). In fact differences in torsional stiffness between the other frames were not statistically significant. Compared to connecting elements that were placed perpendicular to the fixator rings, TSF struts placed in an oblique position would provide additional resistance against translational force along the axis of its body. With six struts distributed in a circular manner, the configuration would make the frame stiff on horizontal plain against loading from any direction. Advantage of conventional IEF over other fixator designs was based on its ability to allow axial motion and resist torsional or translational motions over the bone ends ^10^. Oblique struts of TSF accentuates the pliability in the wire and pin fixation to provide axial compression to stimulate new bone formation, and at the same time resist torsional and translational motions that is detrimental to bone healing. Our findings showed that standard configuration of TSF with 6 obliquely placed struts are able to provide favorable mechanical properties for bone healing.

Stiffness of an external fixator can be improved by application of additional elements to the fixator frame. However, this may provide little benefit to the overall treatment because this may be offset by the added weight, bulk and cost of the device. Improvement in the basic design and configuration of external fixator frame would be more effective to achieve better outcome, and the additional stability provided by obliquely placed connecting elements between fixator rings will have the potential to increase the quality and final outcome in the management of nonunion, bone lengthening and deformity correction. A comparative study on animal and human subjects would be necessary to provide clinical evidence to support these findings.

There are some limitations is our study. Firstly although the PVC tube offers a standardized material for testing, it may not represent the property of bone in clinical practice. Torsional load was measured using a manual method that may have introduced errors. The free play in the TSF frame was not taken into consideration during testing as very little loads are required to take up the free play and thus making testing difficult. A larger sample size utilizing composite resin bone models or cadaveric models may provide more representative values for use in clinical practice. This study also did not consider varying angles of the TSF strut as it has been reported that the TSF frame has some inherent instabilities when the strut angles are less than 30° ^14^.

(Fig. 4)

**Fig. 3a 3b: 3a - Side view of Frame B fixed to the PVC tubes. 3b - Axial view showing Frame D with eccentric mounting of the tube. The half pins are fixed over the anterio-medial aspect of tube .**
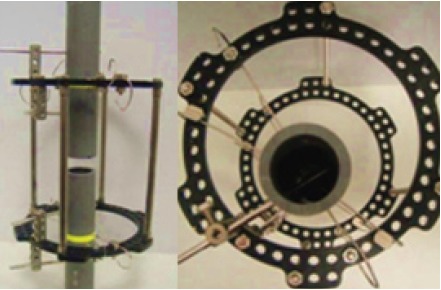


**Fig. 4a 4b: 4a - Image showing a 20mm gap between the tubes during one of the bending load testing. 4b - Image showing the jigs used for bending and torsional loading.**
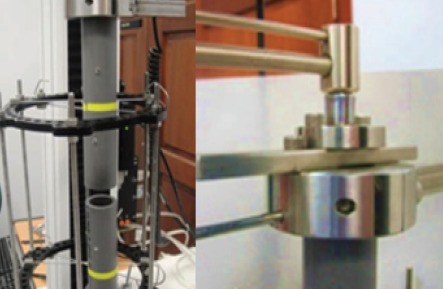


**Fig. 6: Graph showing axial stiffness, anterior, posterior, medial and lateral offset axial stiffness and torsional stiffness of each frame design loaded via bone substitute.**
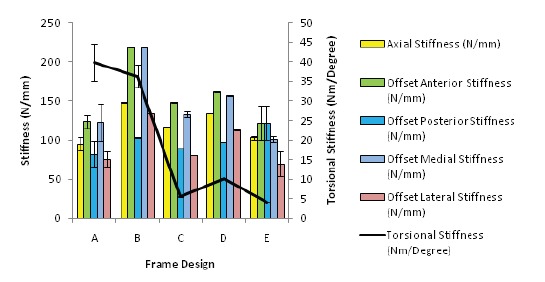


## Conclusion

Standard TSF with 6 oblique struts fixed on to bone model can provide comparable stiffness on axial loading and better stiffness on torsional loading to conventional IEF with 4 threaded rods. The mechanical properties are theoretically favorable for both fracture healing and new bone formation. Changing to stronger hollow connecting bars or increasing the number of threaded rods did not significantly increase the stability against torsional forces. Our findings suggest that TSF may provide a better alternative to conventional IEF as far as mechanical property is concerned.
